# Development and User Testing of an Online Platform to Deliver School-Based Inclusive Physical Education Solutions: Mixed Methods Study

**DOI:** 10.2196/89476

**Published:** 2026-10-07

**Authors:** Sangeetha Mohanraj, Laurie A Malone, Sunkanmi Arogbokun, Lauren Lieberman, Mohanraj Thirumalai

**Affiliations:** 1 SHP Research Collaborative, School of Health Professions, University of Alabama at Birmingham, 701 20th Street South, Birmingham, AL, 35294, United States; 2 Department of Occupational Therapy, School of Health Professions, University of Alabama at Birmingham, Birmingham, AL, United States; 3 Rehabilitation Science, School of Health Professions, University of Alabama at Birmingham, Birmingham, AL, United States; 4 Department of Kinesiology, Sport Studies and Physical Education, SUNY Brockport, Brockport, NY, United States; 5 Division of General Internal Medicine and Population Sciences, Heersink School of Medicine, University of Alabama at Birmingham, 701 19th Street South, ALGEN 580, Birmingham, AL, 35294, United States, +1 2059342206

**Keywords:** students with disabilities, adapted physical activity, inclusive physical education, teacher training, professional development, user testing, usability, mixed methods

## Abstract

**Background:**

Inclusive physical education (PE) plays a vital role in promoting participation and development among students with different abilities. However, many teachers do not have adequate tools to modify PE activities to meet these diverse needs. In addition, parents are essential partners, as their involvement helps reinforce strategies and provide useful information about their children. While online platforms provide a practical way to deliver such solutions, only a few have been intentionally created to support both teachers and parents in implementing inclusive PE learning. To address this gap, we codeveloped an online platform with adapted PE experts that digitizes an inclusion screening tool and delivers personalized, evidence-based inclusion strategies and resources to teachers and parents.

**Objective:**

This study aimed to develop an online platform that provides inclusion strategies for PE teachers and to examine how teachers and parents perceived its usability, acceptability, and overall usefulness using a mixed methods approach.

**Methods:**

A mixed methods design was used to evaluate the platform between September 2024 and March 2025. PE teachers (n=8) and parents of children with disabilities (n=8) were recruited through convenience sampling via national social media outreach and completed individual usability testing sessions via Zoom. Usability was assessed using the System Usability Scale (SUS), the Questionnaire for User Interaction Satisfaction (QUIS), and task performance metrics. Semistructured interviews were conducted to explore user experiences. Quantitative data were analyzed descriptively, while qualitative data were analyzed using codebook thematic analysis, with findings integrated using joint display analysis.

**Results:**

All participants successfully completed the assigned tasks, with a few instances of minor difficulty (14 total errors across 136 task attempts). Participants completed both usability testing and semistructured interviews. Usability and satisfaction scores were high across both groups (SUS: mean 85.63, SD 9.14 for teachers and mean 84.69, SD 12.50 for parents; QUIS: mean 8.52, SD 0.82 for teachers and mean 8.00, SD 1.31 for parents, out of 9). Mixed methods integration showed convergence between positive usability ratings and perceived professional value, while also identifying challenges related to navigation, terminology, learning for first-time users, and mobile interface design.

**Conclusions:**

The online platform provides strong usability and high satisfaction among PE teachers and parents. Future work will involve improving implementation and evaluating its impact on students’ participation outcomes.

## Introduction

Inclusive physical education (PE) aims to ensure that all students, regardless of ability, can participate and benefit. This is especially critical given persistent disparities in physical activity. Children with disabilities consistently engage in less physical activity than their typically developing peers and face higher rates of obesity and health-related complications [1]. National guidelines from the US Department of Health and Human Services (2018) recommend that children engage in at least 60 minutes of physical activity daily to support their physical, mental, and emotional well-being. However, a recent study in Canada found that 91% of children and adolescents with disabilities did not meet daily physical activity guidelines, compared to far lower proportions among their typically developing peers [2]. This inactivity has clear negative consequences for physical, mental, and emotional health, and underscores a critical need for inclusive PE practices that ensure equitable access to physical activity opportunities for all students during the school day.

At the same time, simply placing students with disabilities into general PE classes does not automatically yield positive outcomes. Recent research highlights that inclusive experiences depend on intentional practices and support [3]. Traditional, ableist approaches where ability is measured by performative criteria continue to marginalize students with disabilities and limit participation for all learners [4]. Haegele et al [3] surveyed 99 adapted PE specialists and identified 460 distinct barriers and 473 facilitators to inclusion, spanning seven thematic categories. Notably, they found that teacher-related factors were the most commonly cited barriers, more so than environmental or policy factors. This suggests that the attitudes, training, and strategies of PE teachers largely determine whether an “integrated” class truly feels inclusive. Other researchers have also questioned whether typical integrated PE settings, without additional supports, provide meaningful inclusion [5]. However, Universal Design for Learning (UDL) has been identified as a proactive framework for addressing learner variability in PE. By embedding UDL principles, educators can design lessons that minimize barriers for all students, rather than relying on individual modifications after challenges arise [6]. Research shows that UDL-based approaches not only support students with disabilities but also enhance engagement and outcomes for their peers without disabilities [7,8]. In short, inclusive PE is a complex challenge: it offers clear benefits for students (social interaction, improved fitness, and enhanced well-being), but achieving those benefits requires overcoming significant practical barriers in day-to-day instruction [9].

Unfortunately, many general PE teachers report feeling underprepared to effectively include students with disabilities in their classes. A lack of formal training, limited experience, and insufficient resources contribute to significant barriers in delivering high-quality, inclusive PE [10]. This is consistent with broader findings that many PE teachers feel underprepared to implement inclusive pedagogies, often due to limited exposure to effective strategies and insufficient professional development [11]. Teacher-specific practices such as offering a variety of instructional strategies, adapting equipment and rules, and collaborating with support personnel are essential for reducing environmental barriers and fostering meaningful participation [12]. Teachers frequently cite inadequate support, limited professional development opportunities, and a lack of practical tools as major obstacles to fostering inclusive environments. Alarmingly, over half (54%) of PE teachers report receiving no professional development related to inclusive practices from their school districts [10]. This issue is highlighted in research by Polat et al [13], who conducted a needs analysis with 133 teachers: they concluded that teachers’ knowledge regarding special education students is often insufficient, and educators overwhelmingly desire more training and support to teach inclusively. This lack of professional preparation has been identified as a widespread issue that must be addressed to improve educational outcomes for students with disabilities [14,15], indicating a pressing need to support PE teachers through better training, resources, and peer support.

Current approaches to preparing PE teachers for inclusive practice include preservice coursework, continuing professional development workshops, consultation with adapted PE specialists, and instructional frameworks such as UDL [12,16]. While these approaches provide important foundational knowledge, access is often inconsistent, time-limited, and may not provide ongoing, just-in-time support for teachers managing real-world classroom inclusion challenges [10]. Digital platforms offer an opportunity to extend professional learning by providing scalable, accessible, and on-demand support, particularly for educators who may lack regular access to specialized training resources [13].

In response to this need, the Lieberman/Brian Inclusion Rating Scale for Physical Education (LIRSPE) was developed as a 27-item instrument designed to assess the extent to which PE teachers implement inclusive practices for all students, including those with disabilities, in their programs. The LIRSPE has demonstrated strong reliability and validity [17], making it a valuable tool for evaluating inclusive practices in PE settings. The tool was developed primarily as a practice-oriented self-assessment instrument to support educators in reflecting on and improving their inclusive teaching practices, rather than solely as a research measure. The lack of digital integration constrains data collection, limits opportunities for sharing successful strategies, and reduces its potential as a tool for ongoing professional development. Although the LIRSPE is valuable for assessing inclusive PE practices and identifying areas for improvement, its original static format does not provide embedded implementation guidance or tailored training resources. This is important given evidence that PE teachers often require practical professional development support to effectively implement inclusive practices [16,18]. As digital technologies continue to transform educational practice, there is an opportunity to adapt the LIRSPE into a more accessible and interactive format. A digital platform could enhance its use by supporting real-time feedback, facilitating resource sharing, and promoting sustained improvements in inclusive teaching practices across varied educational contexts.

Parents are important stakeholders in inclusive PE, as they provide valuable insight into their child’s needs, support participation beyond the school environment, and often advocate for appropriate inclusion and accommodations [19]. However, like PE teachers, parents often face similar barriers in accessing clear, actionable guidance for supporting inclusive PE at home and in the community. Including parents in the evaluation of the platform was therefore important to ensure that the resources and recommendations were understandable, relevant, and supportive of collaborative efforts between families and educators to promote inclusive PE participation.

To address these limitations, the present project developed a web-based platform to expand the use of the LIRSPE in supporting inclusive practices in PE. A mobile-friendly digital version of the LIRSPE, accessible via smartphones and tablets, was created, with a self-evaluation interface for teachers and para-PE teachers. In addition, the platform integrated a library of evidence-based solutions to support inclusive instruction. The design of the proposed platform was further informed by UDL, which emphasizes reducing barriers through multiple means of engagement, representation, and action and expression [7-9]. Consistent with these principles, the platform provides inclusion resources in multiple formats, including videos, downloadable documents, and interactive discussion forums, allowing users to access information in ways that align with their needs and preferences. In addition, tailored recommendations generated from users’ LIRSPE responses provide individualized support, aligning the platform with the UDL goal of addressing learner variability and promoting inclusive participation [7-9]. This project followed a 2-phase design. Phase 1 involved the development of the platform, including the digitization of the LIRSPE and the integration of evidence-based inclusion resources, and phase 2 involved a usability evaluation of the platform with PE teachers and parents of children with disabilities. The present paper reports directly on phase 2. Accordingly, this study aimed to examine PE teachers’ and parents’ perceptions of the platform’s usability, acceptability, and overall usefulness using a mixed methods approach.

## Methods

### Development of the System

The web-based platform was built on WordPress, chosen for its robust content management capabilities and flexibility for nontechnical users. To digitize the LIRSPE, a custom software plug-in was developed to create a dynamic survey interface. This feature adapts to the specific user, loading distinct questions for teachers, parents, or students, and using automated logic to calculate scores and assign credits immediately upon submission. Based on these scores, the system triggers the Adapted PE Decision Support Tool, delivering personalized, evidence-based inclusion solutions and recommended resources to the user. Users can also generate and download PDF summaries of their results and completion certificates directly from the dashboard.

To support ongoing professional development and peer interaction, the platform integrates a discussion forum (powered by bbPress) with automated email notifications to alert users of new replies. User access, registration, and role management are handled through a secure interface (Ultimate Member). The visual design used the Divi theme, which was customized to ensure a consistent, accessible layout across devices, enabling intuitive navigation between the survey, results, and resource libraries. Finally, a custom administrative dashboard was built to facilitate data management, enabling the research team to monitor submission dates, analyze demographic trends, and export data for reporting.

### Development of Inclusion Solutions

To develop the recommended solutions provided to participants upon completion of the online LIRSPE, 2 long-standing experts in adapted PE conducted a comprehensive resource review. This process involved a comprehensive search of relevant literature and online content, including YouTube instructional videos, Google searches, and materials from professional organizations such as SHAPE America and Human Kinetics. The experts also incorporated resources they had previously developed and, where gaps were identified, created new materials to address specific areas of need. The platform was designed to identify barriers to inclusive PE and provide personalized, evidence-based solutions and practical resources to improve knowledge, support decision-making, and increase confidence in implementing inclusive practices. This multisource approach ensured that the recommended solutions were both evidence-informed and practically applicable for PE teachers seeking to improve the inclusion of students with disabilities.

### Mixed Methods Research Design

This study examined the strengths and weaknesses of the PE inclusion solutions platform using the International Organization for Standardization (ISO) usability criteria of effectiveness, efficiency, and satisfaction [20]. Acceptability was reflected in participants’ perceptions of the platform’s appropriateness and willingness to engage with or recommend its use, while usefulness referred to the perceived practical value of the platform in supporting inclusive PE decision-making and resource access. A mixed methods approach was used to provide a comprehensive assessment by integrating quantitative and qualitative insights. Specifically, we used the iterative convergent mixed methods design described by Alwashmi et al [21], which involves multiple cycles of concurrent data collection and analysis. This design allowed us to identify and address usability concerns in real time and ensured alignment between research objectives, data generation, analysis, and interpretation. The iterative process involved integrating quantitative usability metrics (task completion time, errors, and questionnaire scores) with qualitative findings from the think-aloud protocol and semistructured interviews. Following each participant session, the research team reviewed the findings, and any technical glitches identified were addressed before the next participant completed testing. More substantial refinements based on recurring feedback are described in the “Results” section. Figure 1 presents the procedural steps followed in this process [21].

**Figure 1.**
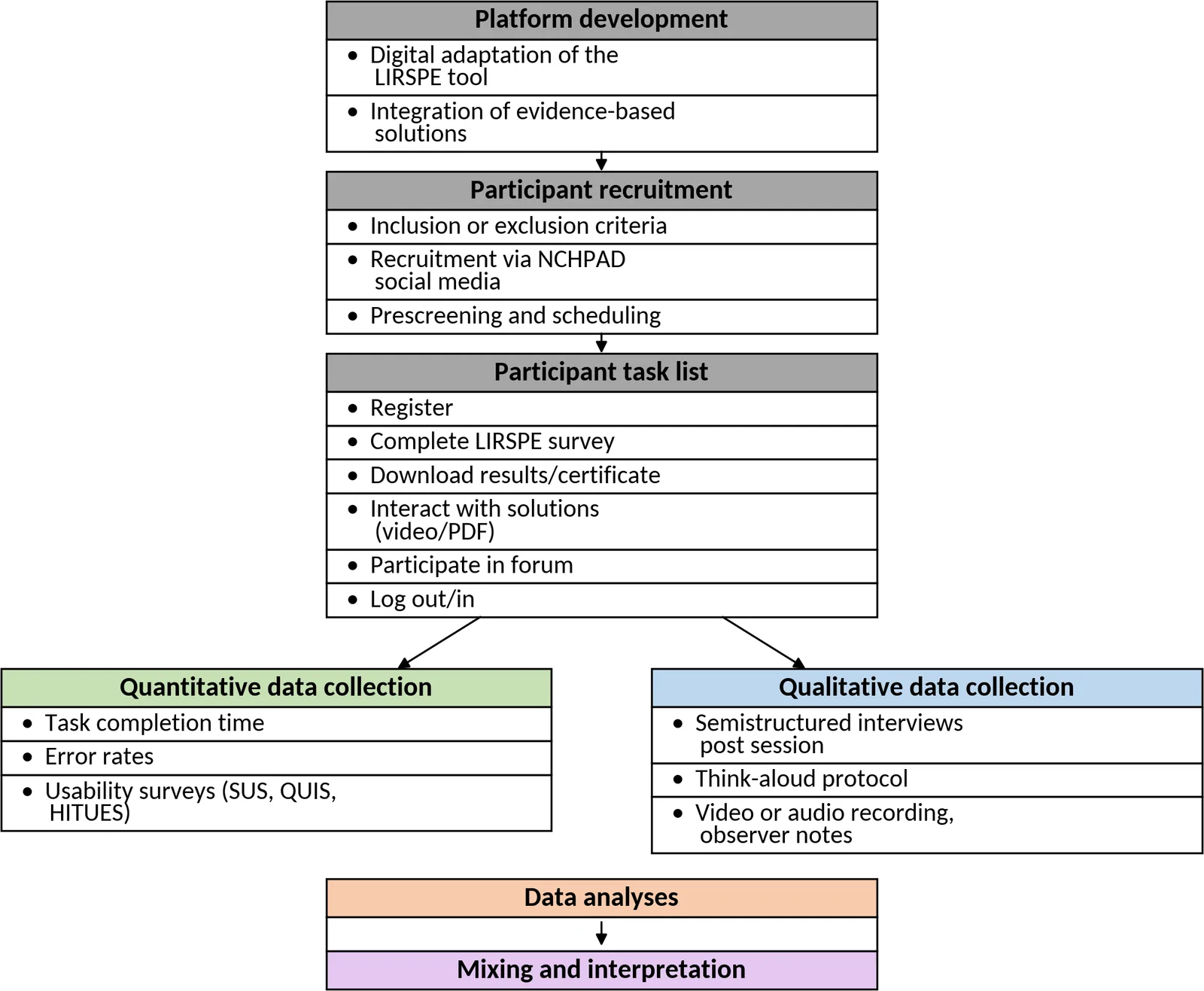
Procedural flow of a mixed methods design for user testing of an online platform to deliver school-based physical education (PE) inclusion solutions. HITUES: Health Information Technology Usability Evaluation Scale; LIRSPE: Lieberman/Brian Inclusion Rating Scale for Physical Education; NCHPAD: The National Center on Health, Physical Activity & Disability; QUIS: Questionnaire for User Interaction Satisfaction; SUS: System Usability Scale.

### Participants

The PE inclusion solutions platform is designed for use by PE teachers and parents of children with disabilities. Therefore, iterative user testing was conducted with a mix of parents and PE teachers. Participants were recruited nationally through the National Center on Health, Physical Activity & Disability (NCHPAD)’s social media site via public posts that included promotional videos and a flyer describing the study; no direct messaging or individualized outreach was used beyond these public posts. Inclusion criteria were the following: (1) aged 18 years or older, (2) fluent in English, (3) able to use a tablet computer, and (4) parent of a child with a disability or working as a PE professional. Recruitment and data collection were conducted between September 2024 and March 2025. A total of 25 individuals were reached during recruitment. Recruitment continued until the target sample of 16 eligible participants (8 PE teachers and 8 parents) was achieved, consistent with recommendations for iterative usability testing [9,22]. Eligibility screening was conducted by a member of the research team (SM). Individuals who did not meet these criteria were excluded. Eligible participants were provided with the study information and scheduled for a 1-hour individual Zoom session. Participants who did not meet the eligibility criteria or declined participation after screening were not enrolled in the study.

### Ethical Considerations

The study was approved by the University of Alabama at Birmingham Institutional Review Board (IRB-300010295). All participants provided informed consent prior to participation. For the online component, oral informed consent was obtained before participants accessed the platform or completed study activities. Participation was voluntary, and all data were deidentified and stored securely to protect participant privacy and confidentiality. Participants received a US $25 gift card upon completion of the study session.

### Data Collection

Data were collected through one-on-one user testing sessions with each participant conducted via Zoom. During those sessions, the researcher provided additional details about the testing process and reiterated that participation was voluntary. The session included navigating various web pages and testing features or functions. The testing session was video recorded (computer screen only) and audio recorded. A research staff member took written notes from their observations of participants during the usability tasks. Participants were asked to complete the following tasks:

- Task 1: register as a PE professional or a parent.
- Task 2: complete the LIRSPE survey and find a solution or recommendation on how to include a child with a disability (imaginary in case they do not have one) in PE activities. Download the results copy and the certificate.
- Task 3a: download the results copy.
- Task 3b: download the certificate.
- Task 4a: from your results, check a solution that needs to be improved and comment on a video.
- Task 4b: from your results, check a solution that needs to be improved and comment on a PDF. Download or print the specific PDF.
- Task 5a: forum—post a response to the existing post “this is a test” and create a new post.
- Task 5b: forum—create a new post.
- Task 6: log out and log in.

The testing involved a “think-aloud protocol” while participants navigated the system [23]. Participants were asked to spontaneously report everything that went through their minds while performing a set of specific tasks. During testing, the system’s efficiency was assessed by task completion time, and its effectiveness was evaluated based on the errors encountered while performing the tasks. System satisfaction was measured using three validated questionnaires: the System Usability Scale (SUS), a 10-item instrument with a 5-point Likert scale assessing overall usability [24]; the Questionnaire for User Interface Satisfaction (QUIS), which evaluates user satisfaction across multiple interface domains using 9-point rating scales [25]; and the Health Information Technology Usability Evaluation Scale (HITUES), a 20-item instrument with 5-point Likert response options assessing impact, usefulness, ease of use, and user control. All 3 questionnaires were self-administered electronically, with participants completing them independently via a Qualtrics survey link shared during the same Zoom session immediately after the usability tasks, rather than having items read aloud by the researcher or completed on paper. Internal consistency for these instruments has been reported as good to excellent in previous validation studies [26]. After completing the surveys, participants took part in a semistructured interview conducted by a member of the research team (SM). The interviews followed a preplanned interview guide (Multimedia Appendix 1) that explored participants’ experiences, perceptions, opinions, and suggestions regarding the platform while allowing flexibility to discuss issues they considered important. Interviews lasted approximately 10 minutes. All recordings were transcribed for qualitative analysis.

### Data Analysis

Qualitative data (think-aloud and interviews) were transcribed using Zoom and cleaned manually for accuracy before being imported into NVivo (Lumivero) for analysis. Data were analyzed using a codebook thematic analysis approach [27,28]. Two researchers (SA and SM) independently coded the transcripts using a shared codebook. The codebook was developed using a hybrid approach: core coding domains (platform navigation, screen layout, terminology, and learning) were derived deductively from the structured usability evaluation framework assessed by the quantitative instruments (SUS, QUIS, and HITUES) and the interview guide, while additional domains, for example, suggestions for the future, were added inductively as they emerged from the transcripts. SA and SM met iteratively to compare codes, resolve discrepancies, and reach consensus on the final coding structure. Rigor was addressed through these study-specific procedures [28]: credibility was supported by dual coding and a consensus process; transferability was supported by detailing the sample (8 PE teachers and 8 parents recruited via NCHPAD), the Zoom-based testing context, and study procedures; dependability was supported by documenting coding decisions and codebook revisions in NVivo; and confirmability was supported by SA and SM checking each code against its source (transcripts). Descriptive analysis (mean, SD and median, IQR) of quantitative data was conducted.

### Iterative Platform Refinement During User Testing

Throughout user testing, the platform was refined iteratively as usability sessions progressed. After each participant session, the research team reviewed the observed usability issues, technical glitches, and participant feedback. Technical problems that affected task completion (eg, navigation errors, delayed system responses, and duplicated forum posts) were corrected before the next participant completed testing whenever feasible. This approach ensured that subsequently identified issues reflected new usability concerns rather than previously recognized technical faults.

## Results

### Participants Demographics

Participants in the PE teacher (n=8) and parent (n=8) groups underwent user testing. Table 1 provides the demographics of the teachers, including their age (mean 41, SD 14 y), job role, grade level taught, highest education level, state of current employment, and years of work experience. Table 2 provides the demographics of the parents of a child with a disability. Iterative modifications to the platform were made throughout testing in response to observed usability issues and participant feedback, as described in the *Methods* section and summarized in Multimedia Appendix 2.

**Table 1.** Physical education (PE) teacher demographics.

ID	Age (y)	Sex	Job role	Grade-level teaching	Highest education	Work state	Years of work experience	Device used for testing
PE1	29	Male	PE teacher in New Hampshire	Elementary	Master's degree	New Hampshire	6	Laptop
PE2	51	Female	PE teacher in New York	Elementary, middle, and high	Master's degree	Pennsylvania	20	Laptop
PE3	40	Female	Adapted PE specialist	Unified PE and sports (inclusion model in her district), and aged 3‐18 years	Adapted PE specialist credentials, MS	California	18	Laptop
PE4	31	Female	PE teacher in South Carolina	Elementary, middle, and high	Master's degree	South Carolina	5	iPhone
PE5	40	Female	N/A^a^	N/A	Master's degree	New York	13	Laptop
PE6	32	Male	Adapted PE specialist	High school (grades 9‐12), adapted and general PE; previously elementary and K-12	Master's degree, pre-doc	Wisconsin	7	Laptop
PE7	72	Male	Prep adapted PE teachers (undergrad and grad)	Not working with schools directly	PhD	Wisconsin	N/A	Laptop
PE8	36	Male	PE teacher	Elementary, 1-year middle school	BS PE	Alabama	8	Laptop

a N/A: not available.

**Table 2.** Parent demographics.

ID	Age (y)	Sex	Job status	Education	Age of the child with disability (y)	Residing state	Device used for testing
P1	52	Female	Part-time physical therapist at an outpatient pediatric clinic	DPT, clinical specialty certification	18 and 15	Maryland	Laptop
P2	54	Female	N/A^a^	Graduate degree in educational technology and leadership	17	Maryland	Laptop
P3	46	Male	Information technologist	College degree	16	Georgia	Laptop
P4	44	Female	Clinical psychologist	PhD	17	Ohio	Laptop
P5	44	Female	Occupational therapist	OT associate	12	Kentucky	Laptop
P6	42	Female	N/A	Master's degree	10	Idaho	Laptop
P7	44	Female	N/A	PhD	10	Oregon	Laptop
P8	35	Female	Medical assistant (nonprofit)	College degree	9	Florida	Laptop

a N/A: not available.

Multimedia Appendix 3 summarizes task completion outcomes, observed difficulties, and errors encountered by PE teachers and parents during usability testing of the PE inclusion solutions platform.

### Effectiveness

Errors during task performance and task completion were used to assess system effectiveness. Overall, both PE teachers and parents were able to complete most assigned tasks with minimal errors (Multimedia Appendix 3). Among PE teachers, the majority of errors occurred during forum interactions, with 4 participants encountering difficulties when responding to existing posts and 2 participants experiencing issues when creating new posts, mainly due to navigation delays or duplicate submissions. Parents similarly completed most tasks successfully, with only isolated difficulties observed in account creation, logging in and out, and commenting on existing forum posts. These findings suggest that the platform effectively supports core tasks, with minor challenges concentrated around interactive navigation features.

### Efficiency

The efficiency of teachers and parents in completing the various tasks is shown in Table 3.

**Table 3.** Time taken for completing the tasks in seconds for physical education (PE) teachers and parents^a^.

Task	PE teachers, mean (SD)	PE teachers, median (IQR)	Parents, mean (SD)	Parents, median (IQR)
Account creation and login	138.75 (87.85)	113.5 (87.8-145.3)	114.63 (66.44)	92.5 (70.5-135.3)
LIRSPE^b^ survey	786.00 (429.47)	805.0 (547.0-923.3)	635.88 (247.71)	636.0 (508.5-870.3)
Download result copy	22.75 (46.46)	6.5 (4.5-9.0)	37.17 (50.26)	15.0 (11.3-30.0)
Download certificate	4.25 (1.92)	4.0 (3.0-5.0)	N/A^c^	N/A
Solution PDF interaction	84.00 (54.79)	70.0 (41.5-105.5)	103.75 (123.30)	38.0 (27.8-133.5)
Video interaction	70.50 (42.54)	58.0 (36.3-86.3)	110.63 (83.15)	111.0 (56.8-140.5)
Forum: comment on existing post	56.13 (27.85)	48.5 (34.5-77.0)	84.50 (75.90)	60.5 (37.5-92.8)
Forum: create new post	81.13 (19.42)	76.5 (72.3-81.8)	63.63 (25.80)	60.0 (45.3-71.8)
Logout and login	25.63 (11.28)	24.5 (21.8-27.0)	29.88 (18.84)	22.5 (15.8-39.0)

a All time measured in seconds.

b LIRSPE: Lieberman/Brian Inclusion Rating Scale for Physical Education.

c N/A: not applicable.

Task completion times during usability testing were used to assess system efficiency. Table 3 presents the task completion times (in seconds) for PE teachers and parents, including both mean (SD) and median (IQR) values to facilitate comparison.

During the think-aloud process, some participants deviated from assigned tasks to provide additional feedback, which may have influenced completion times. Overall, both PE teachers and parents completed simpler tasks efficiently, while more complex tasks requiring interpretation or multiple steps, such as survey completion (PE teachers: mean 786.00, SD 429.47 s and median 805.0, IQR 547.0-923.3 s; parents: mean 635.88, SD 247.71 s and median 636.0, IQR 508.5-870.3 s) and account creation/log-in (PE teachers: mean 138.75, SD 87.85 s and median 113.5, IQR 87.8-145.3 s; parents: mean 114.63, SD 66.44 s and median 92.5, IQR 70.5-135.3 s), took longer. Parents also spent more time on video interaction (mean 110.63, SD 83.15 s and median 111.0, IQR 56.8-140.5 s), suggesting additional navigation demands.

### Satisfaction

Satisfaction scores, as seen in Table 4, were assessed using 3 validated instruments: SUS, HITUES, and QUIS.

**Table 4.** Usability survey scores for teachers and parents.

Instrument	PE teachers, mean (SD)	Parents, mean (SD)
SUS^a^		
Total	85.63 (9.14)	84.69 (12.50)
HI-TUES^b^		
Total	4.60 (0.38)	4.69 (0.23)
Impact	4.83 (0.31)	4.92 (0.24)
Perceived usefulness	4.50 (0.51)	4.76 (0.35)
Perceived ease of use	4.80 (0.35)	4.78 (0.27)
User control	4.33 (0.76)	4.08 (0.79)
QUIS^c^		
Total	8.52 (0.82)	8.00 (1.31)
Overall reaction to software	8.03 (1.59)	8.13 (1.06)
Screen	8.35 (1.08)	7.80 (1.66)
Terminology and system information	8.03 (2.68)	8.10 (1.36)
Learning	9.69 (0.40)	8.63 (1.57)
System capabilities	9.06 (1.24)	6.69 (2.25)

a SUS: System Usability Scale.

b HI-TUES: Health Information Technology Usability Evaluation Scale.

c QUIS: Questionnaire for User Interaction Satisfaction.

PE teachers and parents reported high usability and satisfaction. SUS scores were high for both PE teachers (85.63) and parents (84.69), exceeding the benchmark score of 68. On the HITUES, PE teachers and parents reported similarly high overall scores (4.60 and 4.69, respectively), with the highest ratings in impact (4.83 and 4.92) and perceived ease of use (4.80 and 4.78), while user control received comparatively lower ratings (4.33 and 4.08). QUIS findings also reflected positive satisfaction, with overall mean scores of 8.52 for PE teachers and 8.00 for parents, particularly in learning (9.69 and 8.63), although parents reported lower ratings for system capabilities (6.69), suggesting opportunities for functional refinement.

### Qualitative Findings

Qualitative findings were organized into structured usability evaluation domains derived from the study’s codebook, including platform navigation, screen layout, terminology and learning factors, overall experience with the system, and suggestions for the future.

### Platform Navigation

Many PE educators and guardians commended the system’s intuitive nature for solution retrieval. A professional participant characterized the survey’s organization as “logical and well-categorized.” Nonetheless, some parents advised simplifying access to critical resources, such as survey results or video libraries, to minimize clicks. While certain parents accepted the scrolling, others deemed it excessive. Another participant suggested that “collapsible headings for grouping solutions would mitigate overwhelm.” There was a prevalent request for reducing scroll length through content reorganization or the inclusion of a “jump to” feature.

Both demographic groups experienced challenges in navigating the forums. A participant noted that the platform lacked intuitiveness for posting forum inquiries. One recommendation was to reposition the “Create New Topic” button to the top of the page.

### Screen Layout

A PE instructor observed that disorganized mobile layouts hindered content readability (PE7). Although many parents valued the material’s organization, some raised issues about the text size in PDF formats. Recommendations for enhancement included expanding white space; increasing font size, or text enlargement and screen reading compatibility; adding visual indicators to improve readability and lessen cognitive burden; and more diverse images of kids with disabilities.

### Terminology and Learning Factors

Some PE teachers valued the formal tone while parents appreciated clear language, yet others found the survey wording too complex. An educator criticized the use of advanced terminology, suggesting it alienated nonexperts: “The survey questions used ‘$50 words’ when ‘$5 words’ would do….it felt like it was designed by PhDs for PhDs” (PE1). A parent expressed the need for simpler language, indicating a desire for clarity. Multiple parents recommended adding definitions or tooltips to enhance understanding and accessibility.

### Overall Experience and Suggestions for the Future

#### Overview

Parents acknowledged the system’s value while expressing minor concerns. One parent rated it positively but had doubts about its correctness. Another parent strongly recommended it as transformative for advocacy. When asked for the most favorite feature, all parents mentioned the detailed solutions provided. The PE teachers regarded the platform as a valuable resource for inclusive education. Participants recognized its promise but requested enhancements for better efficiency. Recommendations for improvement included the addition of progress tracking and clearer error messaging to improve user experience. Participants also suggested the following in the future version of the system: resources to buy adaptive equipment, federal laws for each state, a checklist for para-PE teachers, and search options to find solutions without going through the survey.

#### Joint Display

The joint display provides a visual depiction of the integrated qualitative and quantitative (QUIS score) data (Tables 5 and 6).

**Table 5.** Joint display of quantitative and qualitative findings from the Questionnaire for User Interaction Satisfaction (QUIS) among physical education (PE) teachers.

Domain	QUIS, mean (SD)	Qualitative findings	Representative quote	Integration
Overall reaction	8.03 (1.59)	Valuable resource; most PE teachers would recommend the system.	“I’d be more inclined to use this during my prep periods. if I present at a State convention, I would probably use this resource.” (PE6)	High score converges with perceived professional value and willingness to recommend.
Screen	8.35 (1.08)	Layout was generally easy to navigate, but spacing was an issue on mobile.	“Everything was labeled really nicely. The dropdown menus made a lot of sense.” (PE5)	High score converges with positive desktop layout feedback.
Terminology	8.03 (2.68)	Mostly easy to understand, though some wording was viewed as too technical.	“Discussion boards used simple, precise wording anyone could understand.” (PE2)	Qualitative data complements the score.
Learning	9.69 (0.40)	Mixed experiences on user-friendliness and efficient access to PE solutions.	“It’s easy to find and look through things to find stuff.” (PE2)	High score mostly converges with positive learning feedback, though workflow efficiency can improve.
System capabilities	9.06 (1.24)	Content was viewed as reliable; navigation was mostly easy, but forum speed was slow.	“Navigation, it was pretty smooth, pretty easy.” (PE8)	High score converges with content reliability and navigation, with minor technical issues noted.

**Table 6.** Joint display of quantitative and qualitative findings from the Questionnaire for User Interaction Satisfaction (QUIS) among parents of children with disabilities.

Domain	QUIS, mean (SD)	Qualitative findings	Representative quote	Integration
Overall reaction	8.13 (1.06)	Beneficial and supportive; parents would recommend the system.	“The whole idea itself is commendable.” (P3)	High score converges with perceived value and recommendation of the system.
Screen	7.80 (1.66)	Mixed reviews on layout; some found the content visually overwhelming.	“The layout is well-organized, with clear sections and headings.” (P7)	Qualitative findings expand on the lower quantitative score.
Terminology	8.10 (1.36)	Mixed reviews; language was generally clear but may need simplification for some parents.	“The language used in the system is clear and understandable.” (P7)	Qualitative themes expand the score.
Learning	8.63 (1.57)	Mixed experiences on user-friendliness; some users felt unsure during tasks.	“It was very user friendly, easy, not overly complicated.” (P8)	High score converges with positive feedback while leaving room for improvement.
System capabilities	6.69 (2.25)	Mixed reviews on navigation, with suggestions to improve features and speed.	“After submitting a comment, it was difficult to return to the survey results; adding a back button would be helpful.” (P2)	Lower score aligns with navigation and feature-related concerns.

Overall satisfaction was high for both PE teachers (8.03) and parents (8.13), a finding that converges with the universal theme of the system being a “valuable resource.” This high rating is expanded by the qualitative data, indicating satisfaction is rooted in the system’s mission and purpose. Furthermore, both groups found the system exceptionally quick to master (learning between 9.69 and 8.63), yet this still diverges with “mixed experiences” due to functional flaws and complex language.

The most critical finding was the divergence in the system capabilities domain. PE teachers reported a 9.06 score for system capabilities converging with expert-level content trust. In sharp contrast, the parent group scored significantly lower (6.69), which was complemented by qualitative data detailing specific functional failures and navigation. While the PE teachers scored well (8.35) in the screen domain, the parents group scoring lower (7.80) was expanded by complaints of visual clutter that acted as a barrier for the nonexpert group. Regarding the terminologies, for PE teachers, the qualitative data complements the score, confirming that terms are generally appropriate for experts but showing inconsistency (high SD) for a few. For the parents group, the qualitative themes expanded the score, emphasizing the need for simplification and lower reading levels.

#### Explanation of Sections

Participants requested additional information on what to expect before starting the survey (Multimedia Appendix 4), details on what the score means to be moved closer to the score. P5 said, “When directly results show, it is confusing” (Figure 2). The explanation of scoring is provided before the start of the survey. Participants also requested an explanation for the different terms and expansion of abbreviations used in the demographic forms as different jargon is used in different states.

**Figure 2.**
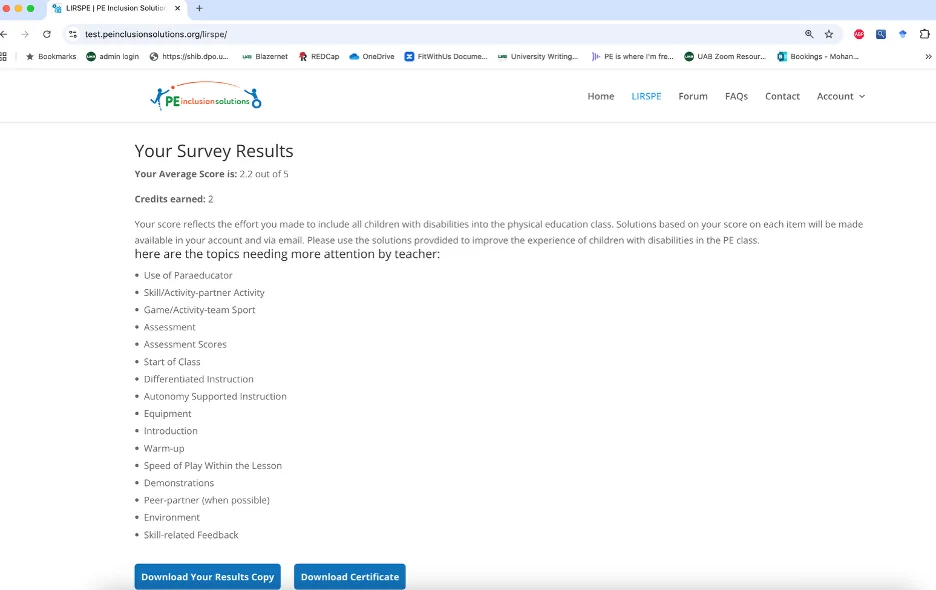
Screenshot of the Lieberman/Brian Inclusion Rating Scale for Physical Education (LIRSPE) survey results display interface. PE: physical education.

#### Forum

Overall, most of the participants in both groups had difficulty with the system speed while performing tasks in the forum. There was confusion in finding the add new topic button. PE1 said, “To add new topic, it takes time to scroll down and find the place to add new topic or difficult to find it.”

#### Demographic Form

Participants had confusion with password setup as the requirements for the password were not provided ahead. Similarly, some of them had difficulty using the DOB (date of birth) calendar option provided.

#### System Refinements Following User Testing

During iterative user testing, several system modifications were made to enhance platform usability (Multimedia Appendix 5); these changes are summarized in Multimedia Appendix 2.

## Discussion

### User Experience: Parents vs Teachers

The study findings suggest that the PE inclusion solution is usable and acceptable for both PE teachers and parents of children with disabilities. Both groups completed simple tasks with ease, while complex, multistep tasks were more difficult for both groups, particularly for parents. This difference across task types suggests that task complexity, rather than the platform’s overall design, was the main source of user difficulty. Furthermore, both groups struggled with tasks involving videos, PDFs, or the downloading of results, suggesting that navigation for resource-based activities is not intuitive. The reason for the difference between groups could be their level of familiarity with PE class activities and the terminology used in the surveys, since PE teachers work with this content regularly while parents typically do not. Some participants also deviated from the tasks, especially during the survey portion of the think-aloud process, and engaged in longer discussions, which may reflect their interest in the content rather than difficulty using the platform. Usability challenges were primarily observed in tasks involving complex navigation and access to platform resources, suggesting targeted rather than widespread issues. This pattern indicates that the platform supports core functionality but would benefit from refinement in more cognitively demanding workflows. This is consistent with established benchmarks indicating that scores above 80 reflect excellent usability.

It is important to note that the LIRSPE survey was originally developed as an assessment tool for PE teachers, with terminology and constructs tailored to professional practice [18]. When digitized for this platform, the tool was extended to parent users without a corresponding adaptation of its content or validation for this new group, and parent-specific use does not appear to have been a formal design consideration during digitization. As a result, some parents in this study expressed confusion or uncertainty regarding specific terms and survey items, which is understandable given the tool’s educator-focused design and may partly explain parents’ comparatively lower system-capability ratings. This suggests that fully meeting parent users’ needs may require a parent-adapted tool that is designed with parent input, rather than the current teacher-focused instrument. This expert-novice gap reflects a well-documented expertise reversal effect, in which design elements that support less experienced users can be redundant or even burdensome for domain experts, and vice versa [29]. It is also consistent with the broader literature on parental involvement in physical education, which highlights that parents engage with PE content and terminology differently than PE professionals given their differing roles and familiarity with the subject [19].

The divergence between positive quantitative ratings and qualitative reports of confusion may reflect differences in what the evaluation measures captured. Standardized usability and usefulness measures assessed participants’ overall perceptions of the platform, whereas qualitative feedback captured specific task-level challenges encountered during direct interaction. Participants may also have valued the platform’s perceived relevance and learning potential despite difficulties with navigation or unfamiliar terminology. This discrepancy is itself a notable finding: parents’ SUS scores were close to those of PE teachers and well above the accepted usability benchmark, yet qualitative data indicated that parents struggled to understand and navigate core survey content. This suggests that standardized usability scales, while useful for benchmarking overall ease of use, may not capture comprehension-level barriers faced by lay users of a tool originally designed for domain professionals. For future dual-audience digital tools, this points to a substantive design implication: usability and comprehension should be treated as distinct constructs and evaluated separately, since a high SUS score alone can mask meaningful gaps in understanding for nonexpert users.

Overall, the platform supports basic tasks but becomes less efficient when workflows require multiple steps or interpretation. Streamlining navigation and simplifying complex tasks would improve efficiency for both groups, with the largest gains for parents. The slow speed of the system could have resulted in errors, especially in the forum.

These findings are consistent with prior research showing that usability, clear navigation, and understandable terminology are important for the adoption of digital educational tools, particularly among users with different levels of expertise [13,21]. While digital platforms can improve access to training and support, design complexity and professional terminology may reduce usability for nonexpert users such as parents. This is consistent with established SUS benchmarks indicating that scores above 80 reflect excellent usability [24], suggesting the platform performs well overall despite the specific challenges noted above.

These findings also align with prior research showing that PE teachers often require additional support and training to implement inclusive practices effectively, and that educational technology adoption is influenced by usability, perceived relevance, and user confidence. The challenges observed among parent users further highlight the importance of designing inclusive digital tools that accommodate users with differing levels of expertise and familiarity with educational terminology [14,15].

This study contributes to the literature by extending LIRSPE beyond assessment into a digital platform that provides tailored inclusion solutions and practical resources. Including both PE teachers and parents also offers insight into how different stakeholder groups engage with inclusive digital tools. This platform may serve as a scalable resource for supporting inclusive PE, particularly where in-person training or specialist guidance is limited. These findings also underscore the importance of designing accessible educational technology for users with varying levels of professional expertise [13].

### Future Research

Future research should evaluate the platform in larger and more diverse populations and assess its effect on inclusive PE knowledge and practice over time. Studies should also examine implementation in real-world school settings, including potential barriers to adoption, teacher and organizational readiness, scalability, sustainability, maintenance and updating of platform content, and integration into teacher professional development programs. In addition, theory-driven frameworks, such as the self-determination theory–based classification of teacher motivational behaviors proposed by Ahmadi et al [30], could be used to better understand how the platform supports autonomy, competence, and relatedness in inclusive PE practice. Mapping the platform’s strategies and digital features onto these motivational behaviors may strengthen the theoretical basis and replicability of future evaluations. This approach could move research beyond usability outcomes toward examining behavioral and instructional mechanisms that influence teachers’ inclusive practices and the sustained implementation of inclusive PE.

### Limitations

This study has several limitations. Although the sample size was appropriate for usability testing, the findings should be interpreted as preliminary and may not be generalizable to broader populations. The inclusion of both PE teachers and parents introduced diverse user perspectives and levels of familiarity with inclusive PE concepts, which may have influenced task performance and interpretation differently. In addition, this study focused on usability, acceptability, and perceived usefulness rather than evaluating the effectiveness of the platform in improving inclusive PE knowledge or practice. Recruitment through NCHPAD’s social media channels may have introduced self-selection bias, favoring individuals already engaged with adapted physical activity resources. The study also did not track long-term classroom implementation or student outcomes, and usability testing was conducted via Zoom rather than in real-world PE settings, which may limit how well the findings reflect actual use.

### Conclusions

This study demonstrates that a web-based platform for delivering school-based PE inclusion solutions is both usable and acceptable to PE teachers and parents, as evidenced by high satisfaction scores and successful task completion across user groups. The integration of quantitative and qualitative user-testing data provided insights into the platform’s strengths, such as its professional value and resource accessibility, as well as areas for improvement, including navigation, terminology, and support for nonexpert users.

These findings reflect users’ perceptions of the platform during initial usability testing and should not be interpreted as evidence that platform use improves teachers’ knowledge, confidence, or inclusive teaching behaviors, or that it leads to improved student participation or educational outcomes. Future research is needed to examine whether sustained use of the platform produces these downstream benefits.

## Supplementary material

10.2196/89476 Multimedia Appendix 1 Script used to guide the participant usability testing sessions, including the introduction, background questions, assigned tasks, and posttest debriefing questions.

10.2196/89476 Multimedia Appendix 2 Summary of key system changes implemented in response to participant feedback and usability errors, along with subsequent testing outcomes following each refinement.

10.2196/89476 Multimedia Appendix 3 Additional results showing raw task performance data and completion-time descriptive statistics for physical education teachers and parents.

10.2196/89476 Multimedia Appendix 4 Screenshots of the platform surveys administered during user testing.

10.2196/89476 Multimedia Appendix 5 Screenshots of the survey interface showing the survey section with visual separation between questions and the updated and reorganized survey results section with clickable links to solutions.

## Abbreviations

DOB: date of birth
HITUES: Health Information Technology Usability Evaluation Scale
ISO: International Organization for Standardization
LIRSPE: Lieberman/Brian Inclusion Rating Scale for Physical Education
NCHPAD: National Center on Health, Physical Activity & Disability
PE: physical education
QUIS: Questionnaire for User Interaction Satisfaction
SUS: System Usability Scale
UDL: Universal Design for Learning
