# Supplementary material for: Development and User Testing of an Online Platform to Deliver School-Based Inclusive Physical Education Solutions: Mixed Methods Study
Source: JMIR Hum Factors. 2026 Oct 7;13:e89476. doi: 10.2196/89476 (PMC13645197; doi:10.2196/89476)
Supplement: Supplementary file 1 [file humanfactors-v13-e89476-s001.docx]

**Appendix**

The PE inclusion solutions website can be accessed at <https://peinclusionsolutions.org/>

**Appendix A: Usability Testing Script**

*Script used to guide the participant usability testing sessions, including introduction, background questions, assigned tasks, and post-test debrief questions.*

**Introduction**

Hello, I am ________________. We are happy to have you participate in our study. We are designing a website for physical education inclusion solutions. We currently have an early design of the system that is ready to be previewed by users like you. In the process of designing this system, we would like to get your input.

In today's session I will ask a few background questions and then will move on to letting you try out the system. We will be audio recording this session to help with notetaking purposes afterwards.

I will first orient you to the system and then let you explore the different portions of website. I will then ask you to perform specific tasks. After completion of those tasks, we will resume with a few more questions to understand how well you like the system and how easy it was to use.

There are no correct or wrong answers to any question, we are only wanting to hear your thoughts and your experience in using our newly designed website. Your input and feedback will be used to improve the website. Anytime during today's session, should you not feel comfortable, please let us know.

Do you have any questions before we start?

**Demographics/Psychographics/Behaviors/Context**

Q1. Can you tell me briefly about yourself?

Probe 1. What do you do?

Probe 2. What is your household composition?

Highest degree?

Q2. On a scale of 1 to 5, where 1 is Nothing at all, and 5 is Very much, how would you rate your confidence/comfort in shopping online?

Why?

Q3. Are you aware of any websites or resources for increasing your ability to include children with disabilities into physical education activities?

Yes/No/Please describe?

Q4. What do you like or dislike about existing solutions for physical education inclusion?

Q5. Have you gone online to see if there are any inclusion options for you as a parent or teacher? If so, how often?

Probe 1. When was the last time you did it?

Probe 2. What tasks did you do?

Probe 3. What systems or apps do/did you use for that?

Probe 4. How was your experience then? Did you encounter any difficulties? Which ones?

Q6. Which of the following statements match how much you know about finding physical education inclusion solutions for children with disabilities?

I know nothing about that.

I know a bit, but I could definitely learn more about it.

I know a lot, there are very few things I don't know well.

I am an expert. I do this regularly.

Q7. What is your thought on using an online system to finding physical education inclusion solutions?

Probe 1: Do you think it would help?

Probe 2: Do you think such a system is possible?

<https://test.peinclusionsolutions.org/>

**Tasks**

Thanks for those answers, now let's go ahead and look at the website. The system is meant to be used by parents, teachers, and para-educators and to assist those individuals in finding solutions for including children with disabilities into physical education activities.

[Five different tasks to be assigned, one at a time]

**Updated Tasks**

Task 1: Register as a PE professional

Task 2: Find a solution/recommendation on how to include a child who uses a wheelchair into PE activities. Download results copy and certificate

Task 3: From your results, check two solutions that needs to be improved and comment on a video and pdf. Download or print the specific pdf

Task 4: Forum — post a response to existing post "this is test"

Task 5: Log out and log in. Create a new post "Test on date"

After each task is successful or unsuccessful, the following questions are asked as appropriate:

Question: I noticed you ___ [did something at a specific moment]. Can you please describe what happened at that moment?

Probe 1: What exactly did you do?

Probe 2: Why did you take that approach?

Question: I noticed you tried interacting with [specific UI element] a couple of times, but nothing happened as a result. Can you tell me what your expectations were at the moment?

Question: How did you find the experience of using the website to complete this task?

Probe 1. How did you find the language used?

Probe 2. How did you find the navigation?

Probe 3. How did you find the layout of the content?

Probe 4. How did you find the amount of scrolling you had to do to complete the task?

Probe 5. How did you find the icons used?

Question: Satisfaction rating: On a scale of 1 to 5, where 1 is not at all easy and 5 is very easy, how easy was it to complete this task?

Probe 1: Why that rating and not some other?

Survey link — <https://uab.co1.qualtrics.com/jfe/form/SV_5p9wdv7QA5gm2b4>

**Post-test**

After all tasks are complete (irrespective of success or failure):

Question 1. How would you describe your overall experience with the system now that you used it to complete a few tasks?

Question 2. What did you like the most about the website? Why?

Question 3. What did you like the least? Why?

Probe: Did you encounter any difficulties as you used the website? Please describe?

[During the tasks, if user had missed out on visual cues on the screen for any task, use the following question in the specific context]

Question 4: As you performed the tasks, did you notice these ___ [captions, headings, menu options, icons, links] in any of the screens you saw?

Probe: What do you understand they relate to?

Probe: Do you think they could help you do some of the tasks you did today? How?

Q5. Now that you have tried our system, what is your thought on using the website to find solutions for including children with disabilities into physical education activities?

Probe 1: Do you think it would help?

Probe 2: Do you think such a system is possible?

Probe 3: Would you recommend this system to anyone?

Q6. What can be improved in the system?

Greenphire giftcard — <https://uab.co1.qualtrics.com/jfe/form/SV_cSHVmjlIR5RZQLI>

**Conclusion**

Thank you very much for taking your time to explore the website and answer all my questions. We appreciate your participation!
