# Supplementary material for: Development and User Testing of an Online Platform to Deliver School-Based Inclusive Physical Education Solutions: Mixed Methods Study
Source: JMIR Hum Factors. 2026 Oct 7;13:e89476. doi: 10.2196/89476 (PMC13645197; doi:10.2196/89476)
Supplement: Supplementary file 2 [file humanfactors-v13-e89476-s002.docx]

**Appendix B: System Refinements and Testing Outcomes**

**B1. System Refinements Made During Iterative User Testing**

| **Changes** | **Feedback** | **Testing Milestone** |
| --- | --- | --- |
| **Corrected duplicate form submission on comment posting** | Clicked submit twice when commenting on an existing forum post, recorded as 1 error | After the initial participant, no further duplicate-submission errors occurred. |
| **Logout button made easier to locate** | Could not find the logout button, recorded as 1 error during Task 6 | After the first participant, no logout-related errors were recorded. |
