# Supplementary material for: Development and User Testing of an Online Platform to Deliver School-Based Inclusive Physical Education Solutions: Mixed Methods Study
Source: JMIR Hum Factors. 2026 Oct 7;13:e89476. doi: 10.2196/89476 (PMC13645197; doi:10.2196/89476)
Supplement: Supplementary file 3 [file humanfactors-v13-e89476-s003.docx]

# **Appendix C: Additional Results Tables**

*Raw task performance data and completion-time descriptive statistics for PE teachers and parents.*

**C1. Summary of Task Completion Outcomes, Difficulties, and Errors**

*Summarizes task completion outcomes, observed difficulties, and errors encountered by PE teachers and parents during usability testing of the PE inclusion solutions platform.*

| **Task** | **PE Teachers (n=8)** | **Parents (n=8)** |
| --- | --- | --- |
| **Account creation/login** | 1 difficulty, 2 errors | 1 difficulty, 1 error |
| **LIRSPE survey** | 0 difficulties, 0 errors | 0 difficulties, 0 errors |
| **Download result copy** | 0 difficulties, 0 errors | 0 difficulties, 0 errors |
| **Download certificate** | 0 difficulties, 0 errors | N/A |
| **Solution PDF interaction** | 1 difficulty, 1 error | 0 difficulties, 0 errors |
| **Video interaction** | 0 difficulties, 0 errors | 0 difficulties, 0 errors |
| **Forum: comment on existing post** | 4 difficulties, 4 errors | 2 difficulties, 3 errors |
| **Forum: create new post** | 2 difficulties, 2 errors | 0 difficulties, 0 errors |
| **Logout/log in** | 0 difficulties, 0 errors | 1 difficulty, 1 error |

**C2. Task Completion Times (Seconds) and Descriptive Statistics**

**PE Teachers**

| **Task** | **PE1** | **PE2** | **PE3** | **PE4** | **PE5** | **PE6** | **PE7** | **PE8** | **Mean** | **SD** |
| --- | --- | --- | --- | --- | --- | --- | --- | --- | --- | --- |
| **Create an account & login** | 81 | 129 | 146 | 98 | 72 | 90 | 349 | 145 | 138.75 | 87.85 |
| **Survey** | 855 | 597 | 1128 | 397 | 122 | 763 | 847 | 1579 | 786 | 429.47 |
| **Download result copy** | 137 | 2 | 6 | 7 | 5 | 15 | 7 | 3 | 22.75 | 46.46 |
| **Download copy of certificate** | 5 | 3 | 8 | 3 | 3 | 5 | 5 | 2 | 4.25 | 1.92 |
| **Results/Solution (Videos)** | 32 | 43 | 73 | 34 | 154 | 114 | 37 | 77 | 70.5 | 42.54 |
| **Results/Solution (PDFs)** | 55 | 184 | 28 | 85 | 92 | 146 | 42 | 40 | 84 | 54.79 |
| **Comment on existing post in the forum** | 44 | 98 | 70 | 98 | 38 | 24 | 53 | 24 | 56.125 | 27.85 |
| **Logout and login** | 26 | 25 | 24 | 15 | 30 | 24 | 51 | 10 | 25.625 | 11.28 |
| **Create new post in the forum** | 77 | 79 | 76 | 90 | 57 | 73 | 70 | 127 | 81.125 | 19.42 |

**Parents**

| **Task** | **P1** | **P2** | **P3** | **P4** | **P5** | **P6** | **P7** | **P8** | **Mean** | **SD** |
| --- | --- | --- | --- | --- | --- | --- | --- | --- | --- | --- |
| **Create an account & login** | 261 | 72 | 66 | 60 | 79 | 134 | 139 | 106 | 114.63 | 66.44 |
| **Survey** | 477 | 868 | 537 | 191 | 735 | 883 | 877 | 519 | 635.88 | 247.71 |
| **Download result copy** | N/A | 15 | 15 | 35 | N/A | 10 | 138 | 10 | 37.17 | 50.26 |
| **Download solution PDF and comment** | 19 | 29 | 38 | 24 | 213 | 362 | 38 | 107 | 103.75 | 123.3 |
| **Watch video and comment** | 23 | 68 | 123 | 5 | 261 | 128 | 99 | 178 | 110.63 | 83.15 |
| **Comment on existing post in the forum** | 63 | 36 | 259 | 38 | 110 | 25 | 87 | 58 | 84.50 | 75.9 |
| **Logout and login** | 60 | 33 | 16 | 57 | 15 | 19 | 26 | 13 | 29.88 | 18.84 |
| **Create new post in the forum** | 92 | 60 | 46 | 110 | 33 | 60 | 43 | 65 | 63.63 | 25.8 |

**C3. Raw Task Performance Data — PE Teachers**

| **Task** | **PE1** | **PE2** | **PE3** | **PE4** | **PE5** | **PE6** | **PE7** | **PE8** |
| --- | --- | --- | --- | --- | --- | --- | --- | --- |
| **Create an account & login** | Easy; 0 | Completed; 0 | Completed; 0 | Easy; 0 | Easy; 0 | Easy; 0 | Difficult; 2 | Completed; 0 |
| **Survey** | Completed; 0 | Completed; 0 | Completed; 0 | Completed; 0 | Completed; 0 | Completed; 0 | Completed; 0 | Completed; 0 |
| **Download result copy** | Completed; 0 | Easy; 0 | Easy; 0 | Easy; 0 | Easy; 0 | Easy; 0 | Easy; 0 | Easy; 0 |
| **Download copy of certificate** | Easy; 0 | Easy; 0 | Easy; 0 | Easy; 0 | Easy; 0 | Easy; 0 | Easy; 0 | Easy; 0 |
| **Results/Solution (Videos)** | Easy; 0 | Easy; 0 | Easy; 0 | Easy; 0 | Completed; 0 | Completed; 0 | Easy; 0 | Easy; 0 |
| **Results/Solution (PDFs)** | Easy; 0 | Difficult; 1 | Easy; 0 | Completed; 0 | Completed; 0 | Completed; 0 | Easy; 0 | Easy; 0 |
| **Forum: post response to "this is test"** | Difficult; 1 | Difficult; 1 | Completed; 0 | Difficult; 1 | Difficult; 1 | Easy; 0 | Completed; 0 | Easy; 0 |
| **Forum: add a new post** | Difficult; 1 | Easy; 0 | Easy; 0 | Easy; 0 | Easy; 0 | Easy; 0 | Easy; 0 | Difficult; 1 |
| **Log out and log in** | Easy; 0 | Easy; 0 | Easy; 0 | Easy; 0 | Easy; 0 | Easy; 0 | Completed; 0 | Easy; 0 |

**C4. Raw Task Performance Data — Parents**

| **Task** | **P1** | **P2** | **P3** | **P4** | **P5** | **P6** | **P7** | **P8** |
| --- | --- | --- | --- | --- | --- | --- | --- | --- |
| **Create an account & login** | Completed; 0 | Completed; 0 | Easy; 0 | Easy; 0 | Easy; 0 | Completed; 0 | Completed; 0 | Difficult; 1 |
| **LIRSPE Survey** | Completed; 0 | Completed; 0 | Completed; 0 | Easy; 0 | Completed; 0 | Completed; 0 | Completed; 0 | Completed; 0 |
| **Download result copy** | N/A; N/A | Easy; 0 | Easy; 0 | Completed; 0 | N/A; N/A | Easy; 0 | Completed; 0 | Easy; 0 |
| **Download solution PDF and comment** | Easy; 0 | Easy; 0 | Easy; 0 | Easy; 0 | Completed; 0 | Completed; 0 | Easy; 0 | Completed; 0 |
| **Watch video and comment** | Easy; 0 | Completed; 0 | Completed; 0 | Easy; 0 | Completed; 0 | Completed; 0 | Completed; 0 | Completed; 0 |
| **Comment on existing post in the forum** | Completed; 0 | Easy; 0 | Completed; 0 | Difficult; 1 | Difficult; 2 | Easy; 0 | Completed; 0 | Easy; 0 |
| **Logout and login** | Difficult; 1 | Easy; 0 | Easy; 0 | Completed; 0 | Easy; 0 | Easy; 0 | Easy; 0 | Easy; 0 |
| **Create new post in the forum** | Completed; 0 | Easy; 0 | Easy; 0 | Completed; 0 | Easy; 0 | Easy; 0 | Easy; 0 | Easy; 0 |
