# Supplementary material for: Development and User Testing of an Online Platform to Deliver School-Based Inclusive Physical Education Solutions: Mixed Methods Study
Source: JMIR Hum Factors. 2026 Oct 7;13:e89476. doi: 10.2196/89476 (PMC13645197; doi:10.2196/89476)
Supplement: Supplementary file 5 [file humanfactors-v13-e89476-s005.docx]

**Appendix E: Updated platform Screenshots after incorporating participant feedback**

*Screenshots of the survey interface, showing (a) the survey section with visual separation between questions, and (b) the updated, reorganized survey results section with clickable links to solutions.*

*(a) Survey section with visual separation between questions.*

*(b) Updated, reorganized survey results section with clickable links for solutions.*
