# Supplementary material for: Development and User Testing of an Online Platform to Deliver School-Based Inclusive Physical Education Solutions: Mixed Methods Study
Source: JMIR Hum Factors. 2026 Oct 7;13:e89476. doi: 10.2196/89476 (PMC13645197; doi:10.2196/89476)
Supplement: Supplementary file 6 [file humanfactors-v13-e89476-s004.docx]

**Appendix D: Platform Survey Screenshots during the User Testing**
